# Social feedback enhances learning in Williams syndrome

**DOI:** 10.1038/s41598-022-26055-8

**Published:** 2023-01-04

**Authors:** Johan Lundin Kleberg, Charlotte Willfors, Hanna Björlin Avdic, Deborah Riby, Martyna A. Galazka, Mona Guath, Ann Nordgren, Claes Strannegård

**Affiliations:** 1 Department of Psychology, Stockholm University, Stockholm, Sweden; 2 Department of Clinical Neuroscience, Centre for Psychiatry Research, Karolinska Institute, Stockholm, Sweden; 3 Department of Molecular Medicine and Surgery, Karolinska Institute, Stockholm, Sweden; 4 Department of Psychology, Centre for Developmental Disorders, Durham University, Durham, UK; 5 Gillberg Neuropsychiatry Centre, Sahlgrenska Academy, University of Gothenburg, Gothenburg, Sweden; 6 Department of Psychology, Uppsala University, Uppsala, Sweden; 7 Department of Clinical Genetics, Karolinska University Hospital, Stockholm, Sweden; 8 Department of Laboratory Medicine, Institute of Biomedicine, University of Gothenburg, Gothenburg, Sweden; 9 Department of Clinical Genetics and Genomics, Sahlgrenska University Hospital, Gothenburg, Sweden; 10 Division of Cognition and Communication, Department of Applied IT, University of Gothenburg, Gothenburg, Sweden

**Keywords:** Motivation, Reward, Social neuroscience, Genetics research, Psychology, Human behaviour

## Abstract

Williams syndrome (WS) is a rare genetic condition characterized by high social interest and approach motivation as well as intellectual disability and anxiety. Despite the fact that social stimuli are believed to have an increased intrinsic reward value in WS, it is not known whether this translates to learning and decision making. Genes homozygously deleted in WS are linked to sociability in the general population, making it a potential model condition for understanding the social brain. Probabilistic reinforcement learning was studied with either social or non-social rewards for correct choices. Social feedback improved learning in individuals with Williams syndrome but not in typically developing controls or individuals with other intellectual disabilities. Computational modeling indicated that these effects on social feedback were mediated by a shift towards higher weight given to rewards relative to punishments and increased choice consistency. We conclude that reward learning in WS is characterized by high volatility and a tendency to learn how to avoid punishment rather than how to gain rewards. Social feedback can partly normalize this pattern and promote adaptive reward learning.

## Introduction

Williams syndrome (WS) is a rare genetic syndrome (prevalence 1: 7500^1^) characterized by strikingly heightened social approach behaviors. Individuals with WS are typically described as “hypersocial”, with a strong social interest, friendliness, and attention to other’s^2–6^. Parallel to this, most individuals with the condition have an intellectual disability^7^ and challenges with social cognition^8^ as well as heightened risk of anxiety^9,10^ and atypical face perception^11,12^. The direct cause is a hemizygous deletion of 25–27 genes at 7q11.23^6^. This locus includes genes implicated in the development of the oxytocin system and brain regions important for the social brain in humans such as the amygdala and the orbitofrontal cortex (OFC)^13–15^. The *GTF2I* and GTF2IRD1 genes typically deleted in WS have been linked to sociability in the general human population^13^ and the social phenotype of WS, an effect that may be mediated by altered oxytocin (OT) reactivity^6,14,16^.

### Social motivation in Williams syndrome

Atypical social motivation is a common facet of neuropsychiatric conditions including autism and depression and may act as a causal mechanism or treatment target^17,18^. In contrast, WS is seemingly a rare example of a condition which leads to *enhanced* rather than reduced social motivation^8^. Studies of WS may therefore contribute to our understanding of sociability and its’ consequences at the genetic, neural, and behavioral level^19^. Importantly, the social phenotype of WS is complex and characterized by enhanced social motivation as well as multiple challenges in social domains, including difficulties with emotion recognition and understanding of other’s mental state^8^. Autistic symptoms are also common^6,20–22^.

### Social motivation and learning

Social motivation is hypothesized to influence learning by modulating the intrinsic reward value of social stimuli^17,23^. In WS, enhanced social motivation has been described as a relative strength. At the same time, social motivation could lead individuals with WS to seek social contacts even if this exposes them to risks^24^. The above theories rely on the assumption that the increased social motivation commonly observed in WS is reflected by the way individuals with the condition learn from the environment. Surprisingly, research testing this assumption is extremely scarce. Preschoolers with WS were found to imitate actions more when performed by a socially engaging as compared to a neutral model^25^. Anecdotal evidence suggests that social feedback may improve classroom learning in WS^26^.

### Social reinforcement learning

One of the most important forms of learning consists of adapting one’s actions to maximize the probability of desired outcomes (rewards). For example, a child may learn through trial and error which behaviors in the playground are most likely to result in positive interactions. Probabilistic reward learning is successfully explained by reinforcement learning models, in which action values are updated by *prediction errors*, or the mismatch between expected and received outcome. Reinforcement learning strategies of individuals or groups can be formalized using computational modeling^27,28^. Reinforcement learning parameters are in turn linked to dissociable brain regions, supporting their feasibility as biomarkers. Striatal dopaminergic neurons signal prediction errors^29^. Brain regions including the amygdala and medial prefrontal cortex seem to represent the subjective value of expected and received outcomes, and the balance between reward seeking and avoidance of aversive outcomes (losses)^23,29^. Prefrontal cortical regions are also implicated in regulation of approach related behaviors. Social rewards (such as positive facial expressions) modulate activity in regions involved in reinforcement learning such as the OFC and the striatum^29–31^. In typically developing populations, social rewards drive reinforcement learning much in the same way as symbolic, appetitive (e.g., food) or monetary rewards^23,29,32^ and with similar effectiveness^30,32,33^ (but see Ref.^34^. However, social feedback may be more effective than non-social feedback in tasks where the stated goal is to understand other’s preferences or mental state^32,35,36^.

Individuals with WS show structural and functional alterations in brain regions involved in reinforcement learning, including amygdala hypoactivation, structural changes in the amygdala and hippocampus, increased functional connectivity between the medial prefrontal cortex and visual cortical regions^6,15,19^. In the absence of formalized modelling, it is difficult to examine whether these alterations correspond to specific atypicalities in reward learning. Here, we report results from the first study examining social feedback effects on probabilistic value learning in WS. We hypothesized that social as compared to non-social feedback, would lead to more optimal learning in WS and that this facilitating effect would be stronger than in typically developing (TD) individuals and individuals with intellectual disability (ID) of other etiology. Cognitive modeling was used to characterize the computational mechanisms underlying reward learning under social as compared to non-social feedback.

## Methods and materials

### Participants

#### Williams syndrome

Participants were recruited from family and patient organizations and habilitation services in Sweden. Initially, 32 individuals expressed interest in participating and 29 attempted the task. Of these, 3 found the task too demanding and did not complete it and one was excluded due to an ongoing psychotic disorder, resulting in a final sample size of *n* = 25 (for age and gender proportion, see Table 1).

**Table 1:** Demographic characteristics.

	WS (*n* = 25)	ID (*n* = 24)	TD (*n* = 56)	Group difference
Age (M (SD) [range])	24.12 (12.26) [7–51]	19.72 (12.51) [6–51]	27.82 (15.88) [6–50]	*p* = 0.160 (WS vs. ID); *p = *0.57 (WS vs. TD) *p* = 0.069 (ID vs. TD)^¥^
Gender (F/M)	11/14	11/13	30/28	all* p* > 0.70^‡^
ABAS General adaptive functioning (M (SD) [range])	57.8 (15.41) [40–93]	66.91 (18.54) [40–104]	–	*p* = 0.215^¥^
ABAS Cognitive functioning (M (SD) [range])	58.2 (16.42) [40–97]	66.55 (16.91) [40–97]	–	*p* = 0.220^¥^
ABAS Social functioning (M (SD) [range])	68.15 (14.47) [50–94]	71.82 (18.64) [50–115]	–	*p* = 756^*¥*^
IQ (M (SD) [range])	56.88 (10.54 [40–79])^η^		102.5 (10.49) [85–115]^±^	*p* < 0.001***^¥^

^¥^Mann–Whitney U; ^‡^Chi-square test; ^±^estimated with Vocabulary subtest from the Wechsler Intelligence Scale for Adults, 4th Edition (WAIS-IV). Based on *n* = 19; ^±^based on WAIS-IV (*n* = 13) or the Wechsler Intelligence Scale for Children, 5th Edition (WISC-5, *n* = 3).

Twenty-two of the 25 individuals with WS completed a larger clinical assessment. Genetic testing showed a typical deletion in all of these participants. A clinical psychologist or psychiatrist conducted a diagnostic interview for DSM-5 diagnoses with the individual and a caregiver using the Mini International Neuropsychiatric Interview (MINI)^37^ and rated the severity of anxiety symptoms using the Clinical Global Impression—Severity (CGI-S^38^), a seven-point scale ranging from “1 = normal, not at all ill” to “7 = among the most extremely ill patients”. For each participant, the highest CGI-S score for any anxiety diagnosis was used as a measure of anxiety severity. For details, see Ref.^10^. Co-occurring diagnoses were ADHD (*n* = 3) and autism (*n* = 4), TICS disorder (*n* = 1). In line with previous studies^9^, 15/22 individuals interviewed with the MINI had an on-going anxiety disorder.

#### Intellectual disability

Inclusion criteria in the ID group was a diagnosis of a rare genetic condition associated with intellectual disability but not hypersociability. Initially, 29 participants expressed interest to participate and 27 attempted the task, of which 3 found it too demanding and did not complete it. Hence, the final ID group included 24 individuals (22q11 deletion syndrome, *n* = 6; Coffin-Siris syndrome^39^ *n* = 10; Fragile X syndrome, *n* = 3, Sotos syndrome, *n* = 4). Co-occurring diagnoses were autism (*n* = 4), ADHD (*n* = 3), epilepsy (*n* = 1), specific language impairment (*n* = 2), and agenesis of the corpus callosum (*n* = 1). One adult individual who had a cerebellar tumor during early childhood was included, but exclusion of this participant did not change any of the results. Age and gender proportion are shown in Table 1.

Although the sample size was too small to allow statistical comparison between syndromes in the ID group, descriptive statistics are reported in the Supplementary materials (Table S1).

Typical controls (henceforth TD) were recruited through advertisements on university web pages, social media, and through addresses collected from the Swedish tax registry. Inclusion criteria were: no ongoing medication with known psychotropic effects, no psychiatric or neurological condition, and no diagnosed or suspected genetic condition. Initially, 65 individuals agreed to participate and completed the task. Of these, 9 participants older than 52 were excluded to create a comparison group within the age range of the WS and ID groups. The final sample size was *n* = 56 (age range 6–51) The overall majority of TD adults (n = 35) had completed a university education. Age and gender proportion are shown in Table 1*.*

### Measures of adaptive and intellectual functioning

Parent ratings of adaptive behavior was collected in the ID and WS groups using the Adaptive Behavior Assessment Scale (ABAS, second edition^40^, *n* = 8, third edition, *n* = 24^41^), a normative sample mean of 100 (SD = 15). No significant group differences were found between the ID or WS groups in general adaptive functioning or in the cognitive or social functioning indices (Table 1). Full-scale IQ was assessed in 16 individuals with WS using the Wechsler intelligence scale for adults, 4th Ed (WISC-IV^42^, *n* = 13) or Wechsler Intelligence Scale for Children, 5th Ed (WISC-V^43^, *n* = 3) depending on the participants’ age.

TD participants were invited to complete screening of cognitive ability using the Vocabulary subtest of the Wechsler Intelligence Scale for Adults, 4th Edition (WAIS-IV) which is highly correlated with full scale IQ (r > 0.90)^42^. Scaled scores were converted to standard scores (population mean of 100 and SD = 15) using the formula *IQ* = 100 + 5*(standard score − 10) and used as a proxy measure for IQ. TD participants screened for IQ (*n* = 19) received an average score of 102.5 (SD = 10.49) which is close to population average.

#### Demographic comparisons

As can be seen in Table 1, groups did not differ significantly in age or gender proportion. The WS and ID group did not differ significantly in parent ratings of adaptive functioning whereas the TD group had higher cognitive ability than the WS group.

The Swedish Ethical Review Authority approved the study, which followed the tenets of the Declaration of Helsinki. Written informed consent was obtained from all participants, and from the parents of participants in the WS and ID groups.

### Task and procedure

**Figure 1:**
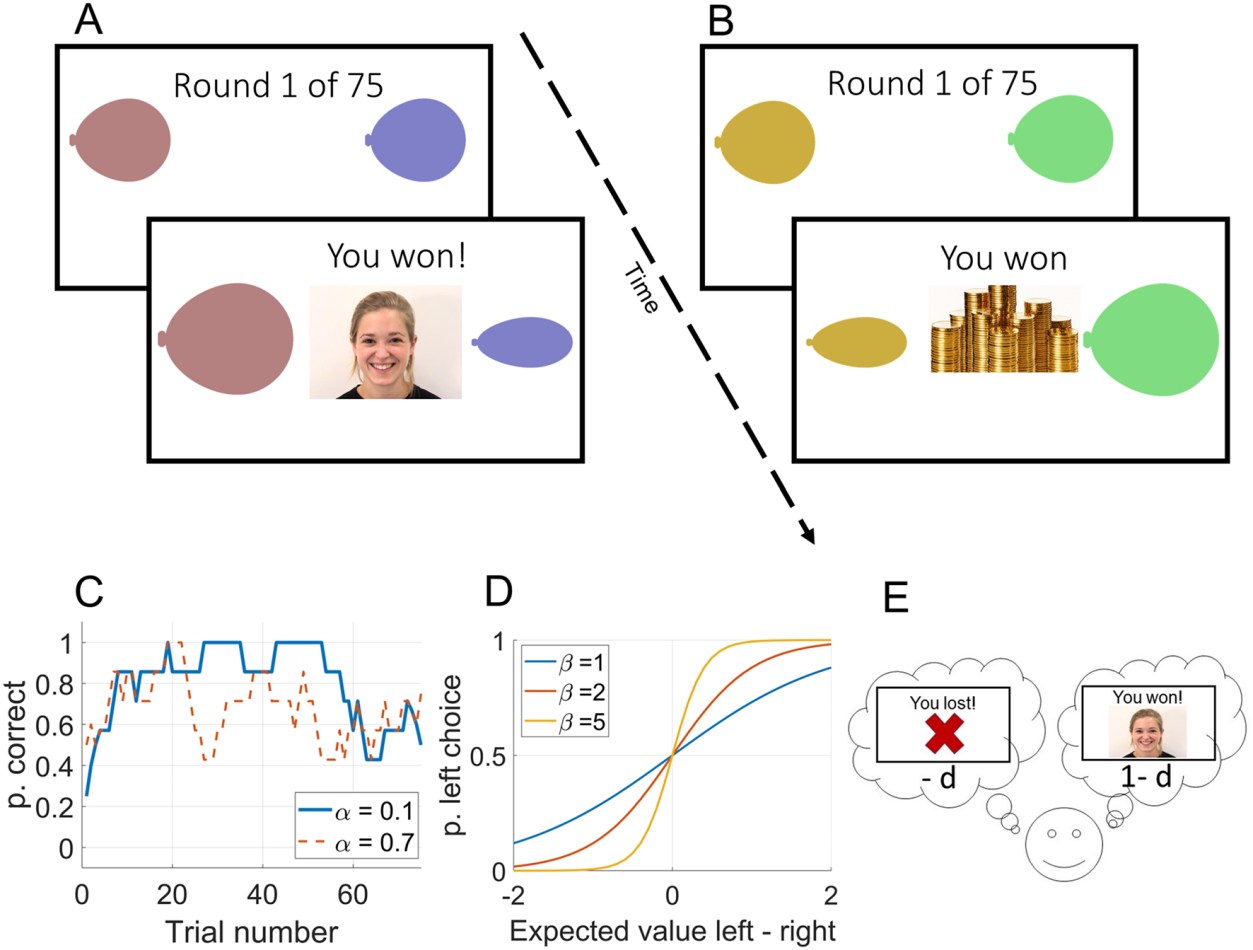
(**A**,**B**) Overview of the experiment. Participants completed the social (**A**) and the non-social feedback condition in counterbalanced order. Each round consisted of 75 trials. Participants were instructed to collect points by choosing between two balloons, and told that one of the options was better. In both conditions, the reward probabilities of the stimuli were 2/3 and 1/3 respectively. Stimulus color and position of the better stimulus (left/right) were counterbalanced between participants and conditions. After selecting one of the two stimuli, participants received either social feedback (an animation of a smiling model, **A**), or non-social feedback (an animation of a pile of gold coins, **B**). (**C–E**) Effects of different values of reinforcement learning parameters. (**C**) Higher values of α increases the degree of updating of action values after each outcome, leading to increased choice volatility. (**D**) Higher values of β (exploitation/exploration balance) leads to more deterministic choices, so that participants prefer the stimulus (left, right) with higher action value. (**D**) The parameter d (loss/reward weight) determines the degree the relative subjective value of losses as compared to wins, so that an agent with d > 0.5 gives higher weight to losses, an agent with d < 0.5 gives higher weight to wins, and d = 0.5 means that equal weight is given to both outcomes.

Participants completed two rounds of a probabilistic reward learning task (described in Fig. 1). Each round consisted of 75 trials where participants chose between two stimuli with a reward probability of 2/3 and 1/3, respectively. A correct choice is defined as selection of the stimulus with the highest reward probability. The 1/3 of trials on which the reward contingencies deviated from the overall pattern (i.e., a correct choice resulted in a loss and an incorrect choice in a gain) were predetermined in six unique reinforcement schedules, which were counterbalanced between participants and conditions.

Twelve participants (WS: n = 2, TD: n = 2, ID: n = 8) completed the task in a research facility, and the rest from home over the internet using a computer or tablet through Pavlovia, a validated system which allows stimulus presentation and reaction time measurement at millisecond precision^44^ Feedback for correct choice was a written message on the screen (“you won!”) and an animation of a smiling woman in the social condition or a pile of gold coins moving towards the participant in the non-social condition. Feedback for incorrect choice was always an animation of the letter X moving towards the participant together with the text “you lost!”. Condition order, reinforcement schedule, and the stimulus associated with the highest reward probability (right, left) was counterbalanced between participants. Animated facial stimuli were taken from the Amsterdam Dynamic Facial Expression Set (ADFES)^45^.

Individuals in the WS and ID groups were assisted by parents or habilitation service personnel who received the same written instructions about how to present the task. In this, they were told that they could assist the participant by reading and explaining the instructions and preparing the testing the environment, but that they should not give any help or advice on how to do the actual task. Instructions were also presented in written form at the screen. The instructions stated that the task was to collect points by choosing between two different balloons. Participants were informed that one of the balloons was better, and that they had to figure out which.

Directly following each round, participants rated their affective experience to winning a point, losing a point, and of seeing the model smile (e.g., the social feedback) and the pile of gold coins (e.g., the non-social feedback) (Fig. 1A,B) on an ascending seven grade Likert scale. All groups rated wins higher than losses, and rated both social and non-social feedback as positive (mean values > 3, see Table 1), indicating that the task was perceived as rewarding. Each round started with four practice trials. To validate the task, 10 participants (WS: n = 2, ID: n = 1, TD, n = 7) repeated the task in a research environment. The small sample size prevents meaningful statistical comparisons, but visualizations of the data indicated highly similar values for choice behavior and computational modeling parameters between measurements (see Supplementary materials).

### Data rejection

Participants who did not explore both options of the task (> 90% choices of one stimulus, ID: *n* = *2,* TD: *n* = 2, WS: *n* = 1) or whose behavior indicated random responses (TD: *n* = *3*, WS: *n* = 1), were excluded from that condition but included in the other condition. Finally, we excluded data from one TD participant in a condition in which more than 50% of reaction times were quicker than 150 MS, indicating inattention.

### Computational modeling

Mathematical modeling allows identification of plausible computational mechanisms underlying observed choice behavior and learning. The method is increasingly used in clinical populations^28,48,49^, but so far not in WS.

We compared model fit to the data of several reinforcement learning models and alternative, non-learning models (e.g., assuming that participants responded at random or switched between choice alternatives regardless of value feedback. Models were further validated through data simulations. Model comparison and parameter estimation were performed through maximum likelihood estimation using the *fminbnd* function in MATLAB. Following previous publications, data were analyzed in two stages^33^. First, for each model, the parameter values which maximized the log likelihood estimate (LLE) of the observed data were selected. In stage two, this procedure was repeated with parameters restrained using Gaussian priors generated in stage one. Specifically, the prior was parametrised with the mean and covariance (joint accross all participants) of the parameters from step 1^33^. This approach has been shown to increase model fit and reduce the risk of extreme parameter values^27,33,48^. For a detailed description, see Supplementary materials.

Parameters of the winning model are described in Fig. 1C–E*.* In short, participants update the expected value of the chosen action V(c) at each trial *t* after seeing the outcome *r* according to the delta rule (e.g., Ref.^28^)

$$V{(c)}_{t+1}={V(c)}_{\begin{array}{c}t\\ \end{array}}+ \alpha \left({r}_{t}-{V\left(c\right)}_{t}\right).$$

Here, $({r}_{t}-{V\left(c\right)}_{t})$ represents the prediction error and α the learning rate. The expected value of the non-chosen action (V(nc)) updated according to the same equation:

$$V{(nc)}_{t+1}={V(nc)}_{\begin{array}{c}t\\ \end{array}}+ \alpha \left(1-{r}_{t}-{V\left(c\right)}_{t}\right).$$

The fact that both actions are updated at each trial was theoretically motivated by the fact that participants were explicitly instructed to learn which of the actions was better, and also provided better fit to the data than models which updated only the chosen options (see Ref.^50^ for a similar model).

Following previous studies^33,51^, the outcome value was determined by the free parameter *d,* so that r = 1 − d if the trial resulted in a win, and r = − d if the trial resulted in a loss. The parameter *d* therefore indices the relative subjective value of rewards and losses, so that the relative weight given to losses is increased at higher values (henceforth referred to as loss/reward balance). Both outcomes are given equal weight if d = 0.5, an agent with d = 1 learns from losses only, and an agent with d = 0 learns only from wins (see Fig. 1E).

Expected values are transformed into choice probabilities with via the softmax function:

$$P\left({c}_{t}\right)=\frac{\mathrm{exp}(\beta V({c}_{t}))}{\mathrm{exp}(\beta V\left({c}_{t}\right))+\mathrm{exp}(\beta V\left({nc}_{t}\right))},$$

where *P*(*c*_*t*_) is the probability of choosing stimulus *c* at trial *t*, V(c_t_) is the expected value of stimulus c, and *V*(*nc*_*t*_) the expected value of the other stimulus at trial t. The parameter $\beta$ ranging from 0 to infinity determines the degree of exploration (Fig. 1B).

### Statistical analysis

Parametric statistics were used since skewness and kurtosis of all variables were within the ± 2 and ± 6 range respectively. Linear mixed effects models (LMM) with random intercepts for participant were used to test interaction effects between group and condition and main effects of group (three levels). Results from LMMs and ANOVAs are identical for a perfectly balanced data set but in contrast to ANOVAs, LMMs can handle unbalanced data (i.e., when a participant has valid data from only one condition) without listwise deletion. P-values were computed using chi square tests, where a model containing the effect of interest was compared to the most complex null model without it^46^. Marginal f2 which represent the proportion of explained variance in the model as compared to the null is reported as effect size^47^.

Significant interaction effects were followed up using t-tests with p-values corrected for multiple comparisons within each variable using the Bonferroni method. Following previous studies^48^, we examined the following metrics: the probability of correct choices (p.correct), the probability to switch after wins (p.win-shift) and losses (p.lose-shift), and the overall proportion of repeated choices p.consistent). Average reaction time (lognormal mean) and reaction time variability (lognormal sigma) was calculated for each participant and condition.

Statistical analyses were conducted using R version 4.1.2 (R Core Team) with the level of statistical significance set to p = 0.05. The study had 80% power to detect medium to large effects (d > 0.4).

## Results

### Reaction times

Significant main effects of group were found on average reaction time and reaction time variability (p < 0.001). Follow-up tests showed that participants with WS and ID were slower and had more variable reaction times than the TD group across conditions (all *p* < 0.001). No main effects of condition or group x condition interactions were found (all *p* > 0.10; see Supplementary Materials, Table S2).

#### Choice behavior

Omnibus models including main effects of group and condition (social, non-social) and group × condition interactions were run for each dependent variable. Results are shown in Table 2. As can be seen, no significant main effects of condition were seen. Main effects of group were found on all choice behavior variables. Bonferroni-corrected pairwise comparisons showed that TD participants made more correct (p.correct) and consistent (p.consistent) choices and were less likely to switch after losses (p.lose-shift) than the WS and ID groups. TD participants were also less likely to switch after wins (p.win-shift) than WS participants, but did not differ from the ID group. No main effects of group were found in pairwise comparisons between WS and ID participants, indicating similar choice behavior when considered across conditions.

**Table 2:** Results from linear mixed effects models (LMMs) of choice behavior variables.

Variable	Effect	χ^2^	df	p	f2	Direction
p.correct	Condition	0.08	1	0.781	< 0.01	
	Group	11.5	2	0.003**	0.08	
	Group (WS vs. ID)	0.04	1	> 0.99^‡^	< 0.01	
	Group (WS vs. TD)	7.18	1	0.021*^,‡^	0.06	WS < TD
	Group (TD vs. ID)	6.32	1	0.036*^,‡^	0.05	ID < TD
	Group × condition	10.18	2	0.006**	0.03	
p.consistent	Condition	0.49	1	0.482	< 0.01	
	Group	33.32	2	< 0.001***	0.23	
	Group (WS vs. ID)	1.21	1	> 0.99^‡^	0.02	
	Group (WS vs. TD)	26.66	1	< 0.001***^,‡^	0.24	WS < TD
	Group (TD vs. ID)	18.29	1	< 0.001***^,‡^	0.17	ID < TD
	Group × condition	21.97	2	< 0.001***	0.03	
p.switch-win	Condition	0.34	1	0.561	< 0.01	
	Group	7.45	2	0.024*	0.05	
	Group (WS vs. ID)	0.32	1	0.572^‡^	0.01	
	Group (WS vs. TD)	6.41	1	0.033*^,‡^	0.06	WS > TD
	Group (TD vs. ID)	3.95	1	0.141^‡^	0.04	
	Group × condition	13.59	2	0.001***	0.03	
p.switch-lose	Condition	0.18	1	0.671	< 0.01	
	Group	51.18	2	< 0.001***	0.33	
	Group (WS vs. ID)	2.52	1	0.336^‡^	0.04	
	Group (WS vs. TD)	38.57	1	< 0.001***^,‡^	0.32	WS > TD
	Group (TD vs. ID)	25.57	1	< 0.001***^,‡^	0.22	ID > TD
	Group × condition	7.08	2	0.029*	0.01	

Significant values are in bold.

*WS* Williams syndrome, *ID* intellectual disability, *TD* typically developed adults. *p < 0.05, **p < 0.01, ***p < 0.001; ^‡^p-values are Bonferroni-corrected for multiple comparisons.

**Figure 2:**
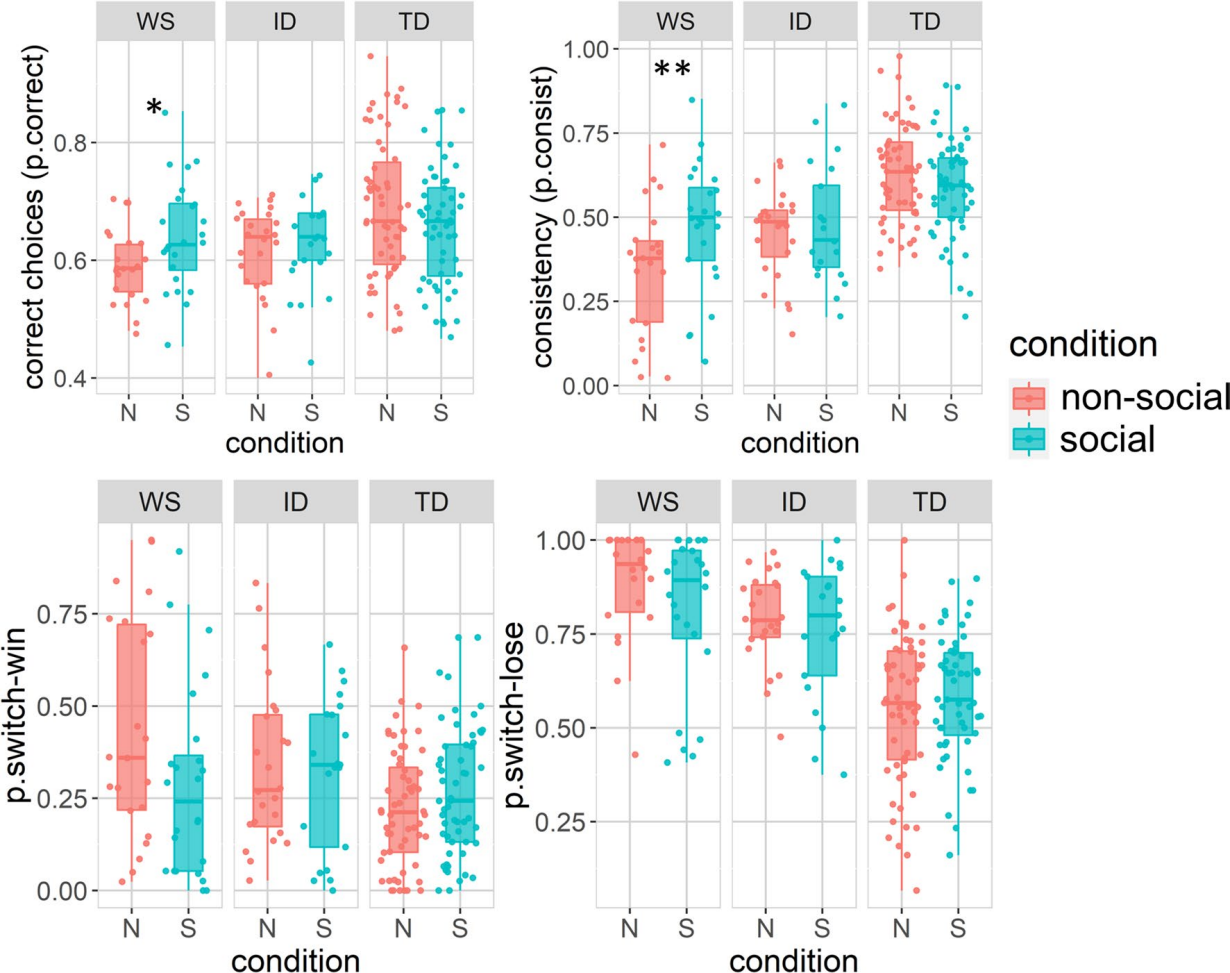
Differences between the social and non-social conditions in choice behavior by group (Stars indicate significant differences from (paired samples t-test, Bonferroni-corrected) for multiple comparisons). Boxplots cover means and 25–75th percentile. *p < 0.05, **p < 0.01. Colored dots show individual participant values.

As predicted, significant group × condition effects were found on all choice behavior variables, indicating that the effects of condition differed between groups. Group × condition effects were followed up with Bonferroni corrected pairwise comparisons within each group separately to address the hypotheses. These are described in Table 3 and Fig. 2. As can be seen, in the social as compared to the non-social feedback condition, the WS group made more correct choices (p = 0.024, d = 0.58), and were more consistent (p = 0.003, d = 0.55), but did not show significant effects on p.win-shift or p.lose-shift. The ID and TD groups showed no significant effects of condition on any of the behavioral indices. For a visualization of the of p.correct over the course of the task, see Supplementary materials, Fig. S2.

**Table 3:** Within-groups comparisons for choice behavior measures.

Measure	Group	Social (M, SD)	Non-social (M, SD)	t	df	P (Bonferroni)	d
p.correct	WS	0.63 (0.09)	0.59 (0.06)	2.91	44	0.024*	0.58
	ID	0.62 (0.08)	0.61 (0.08)	0.74	40	> 0.99	0.19
	TD	0.66 (0.1)	0.68 (0.12)	− 1.68	108	0.297	− 0.24
p.consistent	WS	0.45 (0.19)	0.34 (0.19)	3.99	44	0.003**	0.55
	ID	0.47 (0.17)	0.44 (0.14)	1.19	40	0.741	0.19
	TD	0.59 (0.14)	0.63 (0.14)	− 2.27	108	0.081	− 0.27
p.win-shift	WS	0.32 (0.26)	0.44 (0.3)	− 2.49	44	0.063	− 0.43
	ID	0.32 (0.21)	0.36 (0.23)	− 0.82	40	> 0.99	− 0.16
	TD	0.27 (0.18)	0.23 (0.15)	1.92	108	0.183	0.25
% lose-shift	WS	0.82 (0.2)	0.89 (0.15)	− 2.12	44	0.138	− 0.41
	ID	0.76 (0.18)	0.79 (0.13)	− 0.86	40	> 0.99	− 0.18
	TD	0.58 (0.16)	0.55 (0.19)	1.37	108	0.534	0.17

Significant values are in bold.

*WS* Williams syndrome, *ID* intellectual disability, *TD* typically developed adults. p-values are corrected for multiple comparisons for each variable using the Bonferroni method. *p < 0.05, **p < 0.01, *** p < 0.001.

### Computational modeling parameters

#### Learning rate (α)

**Figure 3:**
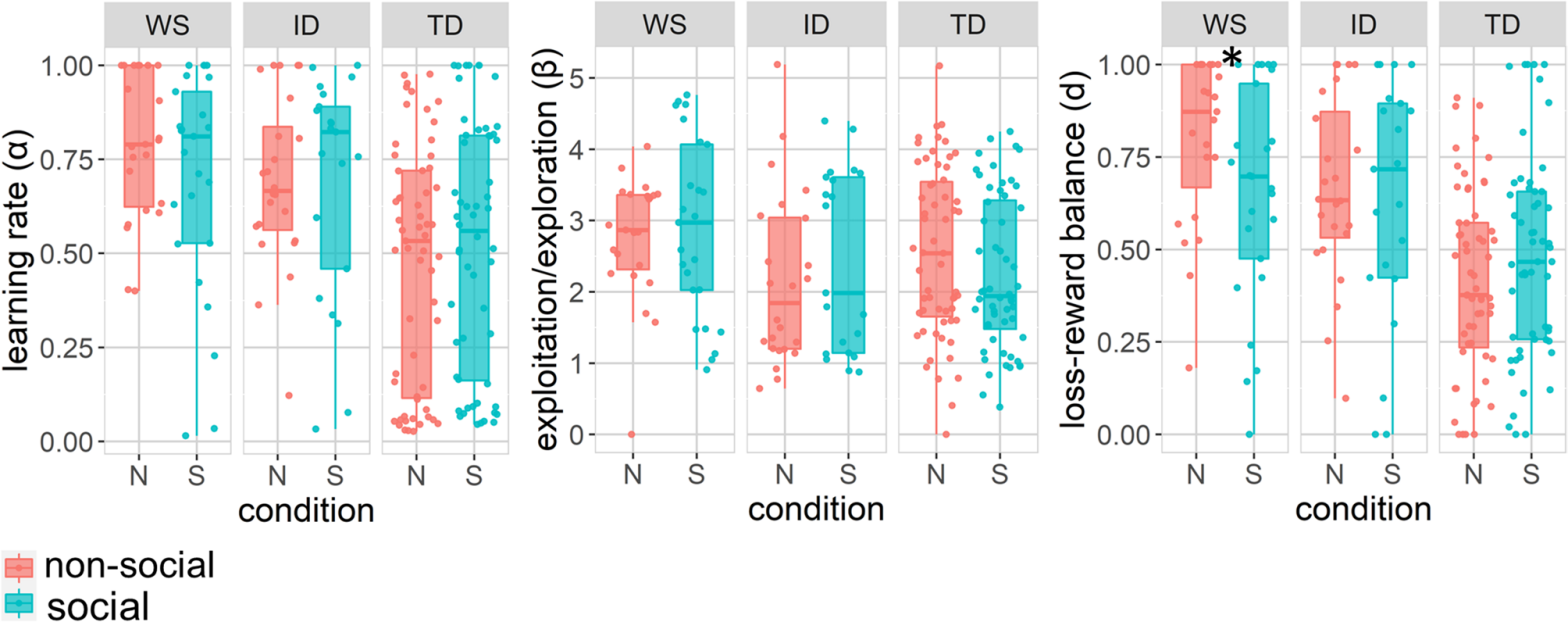
Differences between the social (S) and non-social (N) conditions computational modeling parameters by group. Stars indicate significant differences between conditions (paired samples t-test, Bonferroni-corrected for multiple comparisons). Boxplots cover means and 25–75th percentile. *p < 0.05. Colored dots show individual participant values.

There was no significant main effect of condition (χ^2^ = 2.97, p = 0.226, f2 = 0.018), but a significant effect of group (χ^2^ = 20.71, p < 0.001, f2 = 0.001). The group × condition interaction was not significant (χ^2^ = 2.97, p = 0.226, f2 = 0.018). Pairwise follow-up comparisons showed that learning rate was higher in WS and ID than in TD across conditions, whereas no difference was found between the WS and ID groups (Table 4, Fig. 3).

**Table 4:** Results from linear mixed effects models (LMMs) of computational modeling parameters.

Variable	effect	χ^2^	df	p	f2	Direction
α	Condition	0.02	1	0.892	< 0.01	
	Group	20.71	2	< 0.001***	0.13	
	Group (WS vs. ID)	0.89	1	> 0.90^‡^	0.01	
	Group (WS vs. TD)	15.01	1	0.001**^,‡^	0.12	WS > TD
	Group (TD vs. ID)	9.09	1	0.009**^,‡^	0.08	ID > TD
	Group × condition	2.97	2	0.226	0.01	
β	Condition	0.15	1	0.697	< 0.01	
	Group	4.72	2	0.094	0.03	
	Group × condition	4.02	2	0.134	0.01	
d	Condition	0.01	1	0.939	< 0.01	
	Group	23.62	2	< 0.001***	0.16	
	Group (WS vs. ID)	0.71	1	0.400^‡^	0.01	
	Group (WS vs. TD)	20.53	1	< 0.001***^,‡^	0.18	WS > TD
	Group (TD vs. ID)	11.46	1	0.003**^,‡^	0.10	ID > TD
	Group × condition	10.85	2	0.004**	0.02	

*p < 0.05, **p < 0.01; *** p < 0.001; ‡ p-values are Bonferroni-corrected for multiple comparisons. Significant values are in bold.

#### Exploitation/exploration balance (β)

No significant main or interaction effects were found on *β *(Table 4, Fig. 3).

#### Loss/reward weight (d)

No significant main effect of condition was found (χ^2^ = 0.01, p = 0.939, f2 = 0), but the main effect of group (χ^2^ = 23.62, p = 0, f2 = -0.004) and the group × condition interactions (χ^2^ = 10.85, p = 0.004, f2 = 0.031) were significant. Follow-up comparisons of the main effect showed that *d* was higher in the WS and ID groups than in TD across conditions, (all p < 0.01) whereas the WS and ID groups did not differ (Table 4, Fig. 3). Additional Bonferroni-corrected follow-up tests showed that the WS group had lower loss/reward weight in the social as compared to the non-social condition (t (44) = − 2.84, p = 0.027, d = − 0.44), indicating higher relative sensitivity to social rewards over losses. No significant effects of condition were found in the TD (t (108) = 1.72, p = 0.273, d = 0.25) or ID groups (t (40) = − 1.01, p = 0.969, d = − 0.13). For descriptive statistics see Fig. 3 and Supplementary materials, Table S3).

Linear relationships between loss/reward balance parameter values in the social and non-social conditions and CGI-S anxiety scores in the WS group were tested in a post hoc analysis. These relationships were non-significant (all *p* > 0.30).

#### Relation between reinforcement learning parameters and task performance

Within the WS group, a strong negative correlation between p.correct choices and loss/reward weight in the social condition was found, rs = − 0.88, p < 0.001. This indicates that better task performance after social feedback in WS was mediated through a shift in the relative subjective value of rewards as compared to losses. A significant, although smaller, correlation was also found in the TD group, rs = − 0.40, p = 0.003, but not in the ID group, rs = − 0.31, p = 0.18.

## Discussion

WS is a rare genetic condition with a striking behavioral phenotype characterized by high social motivation, intellectual disability, and high rates of anxiety. This study demonstrates for the first time that social affiliative cues promote optimal decision making (higher probability of correct choices) and modulates reinforcement learning strategies in WS. Social feedback also increased choice consistency in individuals with WS. Computational modeling indicated that this effect could be explained by a shift in the relative subjective values of rewards and punishment towards higher weights given to rewards. Furthermore, at the individual level, lower reward/punishment weights in the social condition were strongly correlated with better task performance (proportion of correct choices) in the WS group.

Notably, the WS group was highly sensitive to losses in both conditions, reflected in reward-loss weights considerably above 0.5 and higher than the TD group (see Fig. 2). Together, these results suggest that in the absence of social feedback, probabilistic learning in WS is biased towards avoiding negative outcomes rather than gaining rewards. Positive social feedback may in turn partly normalize this bias. An interesting question for future longitudinal studies is whether this bias to learn primarily from negative outcomes in WS, is relatively independent of experience or emerges through interaction with the environment.

### Social feedback affects loss/reward balance in WS

The WS group did not show an effect of social as compared to non-social feedback on learning rate or exploitation/exploration balance. Instead, social feedback affected the subjective balance between losses and rewards. That is, the beneficial effects of social feedback seem to operate by increasing the relative valuation or rewards versus losses rather than by updating of action values per se. This is in line with theories derived from autism research which suggests that the influence of social motivation on learning goes through an increase in salience of social stimuli^17,18^. Our results are therefore consistent with the idea that social motivation is increased in WS. Highly volatile performance and fluctuating attention is common in WS. Our results indicate that social feedback may be feasible as a means of reducing these difficulties^52^.

In contrast to WS, the ID group showed no clear effects of feedback type on behavioral measures or reinforcement learning parameters, demonstrating that the effects seen in WS are not explained by ID per se. Notably, the effects of social feedback seen in WS were also absent in the TD group in line with previous studies^32,33,35^. In this group, social as compared to non-social feedback reduced the likelihood of repeating a successful choice but did not affect the overall proportion of correct choices or reinforcement learning strategies. These findings again suggest that social feedback has specific effects of reinforcement learning in WS, which are not seen in TD or ID. Previous research in TD has typically shown that social feedback enhances learning to a similar degree as symbolic non-social rewards, although results are somewhat mixed^30,35,36^. For example, one study reported worse probabilistic learning following social than non-social rewards^30^. Improved probabilistic learning following social feedback was reported in a number of studies where tasks were presented as being about learning others’ preferences or mental states^32,34,36^. Since mental state attribution is challenging for many individuals with WS^53^, an interesting area for future studies is whether facilitating effects of social feedback would extend to this type of task.

Altered reinforcement learning is commonly seen in anxiety disorders and may contribute to their etiology and symptom maintenance. Particularly, anxious populations were found to be more sensitive to losses than controls^52,53^. Given the high prevalence of anxiety disorders in WS, we speculate that the increased loss sensitivity observed in WS may be a risk factor for the development of anxiety disorders. An interesting question for future studies is how social and non-social feedback for losses would affect reinforcement learning in WS.

### Limitations

Some limitations should be mentioned. Sample size in the WS and ID groups was small. The study data were largely collected online. While our results suggests that this is feasible in populations with rare genetic conditions and ID, a limitation of the study is the lack of exact control over the settings in which participants completed the task. However, it should be noted that data collected online and in the lab was highly similar. An additional limitation is that the small sample size in the ID group did not allow formal statistical comparisons between the included conditions (22q11 deletion syndrome, Fragile X syndrome, Coffin-Siris syndrome, and Sotos syndrome). Despite these limitations, the current study contributes to our understanding of WS and the extent to which the previously described social approach motivation in the condition generalizes to learning.

## Supplementary Information

Supplementary Information.

## Data Availability

Anonymized data will be made available to researchers upon reasonable request.

## References

[1] Stromme P, Bjornstad PG, Ramstad K (2002). Prevalence estimation of williams syndrome. J. Child. Neurol..

[2] Järvinen A, Korenberg JR, Bellugi U (2013). The social phenotype of Williams syndrome. Curr. Opin. Neurobiol..

[3] Jones W, Bellugi U, Lai Z, Chiles M, Reilly JS, Lincoln AJ, Adolphs R (2000). II. Hypersociability in Williams Syndrome. J. Cogn. Neurosci..

[4] Ng R, Järvinen A, Bellugi U (2014). Toward a deeper characterization of the social phenotype of Williams syndrome: The association between personality and social drive. Res. Dev. Disabil..

[5] Sampaio A, Belsky J, Soares I, Mesquita A, Osório A, Gonçalves ÓF (2018). Insights on social behavior from studying Williams syndrome. Child Dev. Perspect..

[6] Kozel BA, Barak B, Kim CA, Mervis CB, Osborne LR, Porter M, Pober BR (2021). Williams syndrome. Nat. Rev. Dis. Primers.

[7] Miezah D, Porter M, Batchelor J, Boulton K, Campos Veloso G (2020). Cognitive abilities in Williams syndrome. Res. Dev. Disabil..

[8] van Herwegen J, Smith TJ, Dimitriou D (2015). Exploring different explanations for performance on a theory of mind task in Williams syndrome and autism using eye movements. Res. Dev. Disabil..

[9] Royston R, Howlin P, Waite J, Oliver C (2017). Anxiety disorders in Williams syndrome contrasted. J. Autism. Dev. Disord..

[10] Willfors C, Riby DM, van der Poll M, Ekholm K, Avdic Björlin H, Kleberg JL, Nordgren A (2021). Williams syndrome: On the role of intellectual abilities in anxiety. Orphanet. J. Rare Dis..

[11] Kleberg, J. L., Riby, D., Fawcett, C., Björlin Avdic, H., Frick, M. A., Brocki, K. C. *et al.* Williams syndrome: reduced orienting to other’s eyes in a hypersocial phenotype. *J. Autism Dev. Disord.* 1–12 (2022). 10.1007/s10803-022-05563-6 PMC9020553 35445369

[12] D’Souza D, Cole V, Farran EK, Brown JH, Humphreys K, Howard J (2015). Face processing in Williams syndrome is already atypical in infancy. Front. Psychol..

[13] Crespi BJ, Procyshyn TL (2017). Williams syndrome deletions and duplications: Genetic windows to understanding anxiety, sociality, autism, and schizophrenia. Neurosci. Biobehav. Rev..

[14] Barak B, Zhang Z, Liu Y, Nir A, Trangle SS, Ennis M (2019). Neuronal deletion of Gtf2i, associated with Williams syndrome, causes behavioral and myelin alterations rescuable by a remyelinating drug. Nat. Neurosci..

[15] Barak B, Feng G (2016). Neurobiology of social behavior abnormalities in autism and Williams syndrome. Nat. Neurosci..

[16] Procyshyn TL, Spence J, Read S, Watson NV, Crespi BJ (2017). The Williams syndrome prosociality gene GTF2I mediates oxytocin reactivity and social anxiety in a healthy population. Biol. Lett..

[17] Bottini S (2018). Social reward processing in individuals with autism spectrum disorder: A systematic review of the social motivation hypothesis. Res. Autism Spectr. Disord..

[18] Chevallier C, Kohls G, Troiani V, Brodkin ES, Schultz RT (2012). The social motivation theory of autism. Trends Cogn. Sci..

[19] Meyer-Lindenberg A, Mervis CB, Faith Berman K (2006). May): Neural mechanisms in Williams syndrome: A unique window to genetic influences on cognition and behaviour. Nat. Rev. Neurosci..

[20] Glod M, Riby DM, Rodgers J (2019). Short report: Relationships between sensory processing, repetitive behaviors, anxiety, and intolerance of uncertainty in autism spectrum disorder and Williams syndrome. Autism Res..

[21] Klein-Tasman BP, Phillips KD, Lord C, Mervis CB, Gallo FJ (2009). Overlap with the autism spectrum in young children with Williams syndrome. J. Dev. Behav. Pediatr..

[22] Klein-Tasman BP, van der Fluit F, Mervis CB (2018). Autism spectrum symptomatology in children with Williams syndrome who have phrase speech or fluent language. J. Autism Dev. Disord..

[23] Olsson A, Knapska E, Lindström B (2020). The neural and computational systems of social learning. Nat. Rev. Neurosci..

[24] Ridley, E., Riby, D. M., Leekam, S. R. A cross-syndrome approach to the social phenotype of neurodevelopmental disorders: Focusing on social vulnerability and social interaction style. (2020). 10.1016/j.ridd.2020.103604. 10.1016/j.ridd.2020.103604 32142968

[25] Vivanti G, Hocking DR, Fanning P, Dissanayake C (2016). Social affiliation motives modulate spontaneous learning in Williams syndrome but not in autism. Mol. Autism.

[26] Reis SM, Schader R, Milne H, Stephens R (2003). Music & minds: Using a talent development approach for young adults with Williams syndrome. Except. Child..

[27] Daw, N. D. Trial-by-trial data analysis using computational models. In *Decision Making, Affect, and Learning: Attention and Performance XXIII*. (eds. Delgado, M. R., Phelps, E. A., Robbins, T. W.) (Oxford University Press, 2011).

[28] Zhang L, Lengersdorff L, Mikus N, Gläscher J, Lamm C (2020). Using reinforcement learning models in social neuroscience: Frameworks, pitfalls and suggestions of best practices. Soc. Cogn. Affect Neurosci..

[29] Ruff CC, Fehr E (2014). The neurobiology of rewards and values in social decision making. Nat. Rev. Neurosci..

[30] Lin A, Adolphs R, Rangel A (2012). Social and monetary reward learning engage overlapping neural substrates. Soc. Cogn. Affect. Neurosci..

[31] Ferdinand NK, Hilz M (2020). Emotional feedback ameliorates older adults’ feedback-induced learning. PLoS One.

[32] Frey AL, Frank MJ, McCabe C (2019). Social reinforcement learning as a predictor of real-life experiences in individuals with high and low depressive symptomatology. Psychol. Med..

[33] Hurlemann R, Patin A, Onur OA, Cohen MX, Baumgartner T, Metzler S (2010). Behavioral/systems/cognitive oxytocin enhances amygdala-dependent, socially reinforced learning and emotional empathy in humans. J. Neurosci..

[34] Gorlick MA, Giguère G, Glass B, Nix BN, Mather M, Maddox WT (2013). Attenuating age-related learning deficits: Emotional valenced feedback interacts with task complexity. Emotion.

[35] Colombo M, Stankevicius A, Seriès P (2014). Benefits of social vs. non-social feedback on learning and generosity. Results from the Tipping Game. Front. Psychol..

[36] Sheehan DV, Lecruier Y, Sheehan KH, Amorim P, Juris J, Willer E (1998). The Mini-International Neuropsychiatric Interview (M.I.N.I.): The development and validation of a structured diagnostic psychiatric interview for DSM-IV and ICD-10.—PsycNET. J. Clin. Psychiatry.

[37] Zaider TI, Heimberg RG, Fresco DM, Schneier FR, Liebowitz MR (2003). Evaluation of the Clinical Global Impression Scale among individuals with social anxiety disorder. Psychol. Med..

[38] Sluijs PJ, Jansen S, Vergano SA, Adachi-Fukuda M, Alanay Y, AlKindy A (2022). The ARID1B spectrum in 143 patients: From nonsyndromic intellectual disability to Coffinâ€“Siris syndrome. Genet. Med..

[39] Harrison, P., Oakland, T. *Adaptive Behavior Assessment System—Second Edition (ABAS-II)* (Harcourt Assessment, 2003).

[40] Harrison, P., Oakland, T. *Adaptive Behavior Assessment System, 3d.Ed. (ABAS-3)* (Western Psychological Services, 2015).

[41] Wechsler, D. *Wechsler Adult Intelligence Scale—Fourth Edition*. (Pearson, 2008).

[42] Wechsler D (2014). Wechsler Intelligence Scale for Children.

[43] Bridges D, Pitiot A, MacAskill MR, Peirce JW (2020). The timing mega-study: Comparing a range of experiment generators, both lab-based and online. PeerJ.

[44] van der Schalk J, Hawk ST, Fischer AH, Doosje B (2011). Moving faces, looking places: Validation of the Amsterdam Dynamic Facial Expression Set (ADFES). Emotion.

[45] Baayen RH, Davidson DJ, Bates DM (2008). Mixed-effects modeling with crossed random effects for subjects and items. J. Mem. Lang..

[46] Nakagawa S, Cuthill IC (2007). Effect size, confidence interval and statistical significance: A practical guide for biologists. Biol. Rev..

[47] den Ouden HEM, Daw ND, Fernandez G, Elshout JA, Rijpkema M, Hoogman M (2013). Dissociable effects of dopamine and serotonin on reversal learning. Neuron.

[48] Guath M, Kleberg JL, Weis J, Widegren E, Frick M, Möller S (2023). Pupil dilation during negative prediction errors is related to brain choline concentration and depressive symptoms in adolescents. Behav. Brain Res..

[49] Klein TA, Ullsperger M, Jocham G (2017). ARTICLE Learning relative values in the striatum induces violations of normative decision making. Nat. Commun..

[50] Gold JM, Waltz JA, Matveeva TM, Kasanova Z, Strauss GP, Herbener ES (2012). Negative symptoms and the failure to represent the expected reward value of actions behavioral and computational modeling evidence. Arch. Gen. Psychiatry.

[51] Shalev N, Steele A, Nobre AC, Karmiloff-Smith A, Cornish K, Scerif G (2019). Dynamic sustained attention markers differentiate atypical development: The case of Williams syndrome and Down’s syndrome. Neuropsychologia.

[52] Pike AC, Robinson OJ (2022). Reinforcement learning in patients with mood and anxiety disorders vs control individuals: A systematic review and meta-analysis. JAMA Psychiat..

[53] Mineka S, Zinbarg R (2006). A contemporary learning theory perspective on the etiology of anxiety disorders: It’s not what you thought it was. Am. Psychol..

