# Supplementary material for: Social feedback enhances learning in Williams syndrome
Source: Sci Rep. 2023 Jan 4;13:164. doi: 10.1038/s41598-022-26055-8 (PMC9813264; doi:10.1038/s41598-022-26055-8)
Supplement: Supplementary file 1 — Supplementary Information. [file 41598_2022_26055_MOESM1_ESM.docx]

Supplementary Materials

Descriptive statistics in Fragile X, Coffin-Siris syndrome, Sotos syndrome, and 22q11 deletion styndrome

Descriptive statistics for behavioral measures and modeling parameters in the ID group split by syndrome are shown in *Table S1*. Due to the small sample sizes, no inferential statistical analyses were performed. Cohen’s d is shown as a descriptive measure of between-groups differences.

*Table S1. Behavioral measures and modeling parameters in Fragile X (n = 2), Coffin-Siris syndrome (n = 9), 22q11 deletion syndrome (n = 6), and Sotos syndrome (n = 4)*

|  |  | **Social condition±** | | **Non-social condition** | | |
| --- | --- | --- | --- | --- | --- | --- |
| **Variable** | **Group** | M (sd) | Md (mad) | M (sd) | Md (mad) | Cohen’s d |
| **%correct** |  |  |  |  |  |  |
|  | FXS | 0.64(0.11) | 0.65(0.03) | 0.65(0.12) | 0.65(0.04) | -0.26 |
|  | CS | 0.62(0.07) | 0.57(0.09) | 0.61(0.04) | 0.56(0.1) | 0.53 |
|  | 22q11 | 0.64(0.1) | 0.65(0.06) | 0.68(0.02) | 0.67(0.04) | -0.17 |
|  | Sotos | 0.61(0.01) | 0.63(0.04) | 0.61(0.01) | 0.63(0.04) | -0.95 |
| **%consistency** |  |  |  |  |  |  |
|  | FXS | 0.64(0.15) | 0.54(0.06) | 0.65(0.2) | 0.51(0.02) | 0.95 |
|  | CS | 0.47(0.19) | 0.43(0.17) | 0.41(0.11) | 0.45(0.17) | 0.23 |
|  | 22q11 | 0.44(0.13) | 0.47(0.1) | 0.43(0.13) | 0.49(0.08) | -0.22 |
|  | Sotos | 0.32(0.1) | 0.42(0.12) | 0.32(0.1) | 0.46(0.06) | -0.87 |
| **%win-shift** |  |  |  |  |  |  |
|  | FXS | 0.06(0.05) | 0.18(0.2) | 0.05(0.03) | 0.11(0.12) | -0.93 |
|  | CS | 0.38(0.21) | 0.41(0.25) | 0.45(0.14) | 0.39(0.24) | -0.1 |
|  | 22q11 | 0.3(0.18) | 0.3(0.18) | 0.33(0.15) | 0.28(0.21) | -0.03 |
|  | Sotos | 0.46(0.19) | 0.33(0.22) | 0.46(0.19) | 0.25(0.07) | 0.64 |
| **%lose-shift** |  |  |  |  |  |  |
|  | FXS | 0.67(0.23) | 0.76(0.13) | 0.75(0.15) | 0.76(0.2) | -0.47 |
|  | CS | 0.71(0.19) | 0.75(0.14) | 0.75(0.2) | 0.79(0.12) | -0.26 |
|  | 22q11 | 0.84(0.14) | 0.82(0.09) | 0.86(0.15) | 0.78(0.05) | 0.17 |
|  | Sotos | 0.93(0.02) | 0.87(0.08) | 0.93(0.02) | 0.88(0.05) | 1.26 |
| **α** |  |  |  |  |  |  |
|  | FXS | 0.63(0.2) | 0.6(0.44) | 0.6(0.2) | 0.67(0.48) | 0.11 |
|  | CS | 0.6(0.37) | 0.74(0.13) | 0.78(0.28) | 0.72(0.1) | -0.55 |
|  | 22q11 | 0.85(0.08) | 0.72(0.27) | 0.84(0.12) | 0.61(0.37) | 0.74 |
|  | Sotos | 0.69(0.43) | 0.59(0.16) | 0.69(0.46) | 0.55(0.1) | 0.34 |
| **β** |  |  |  |  |  |  |
|  | FXS | 3.31(1.19) | 2.89(0.45) | 3.66(0.92) | 3.07(0.23) | 0.52 |
|  | CS | 2.35(1.22) | 1.46(0.59) | 2.44(1.72) | 1.32(0.54) | 0.98 |
|  | 22q11 | 1.99(1.23) | 2.27(1.37) | 1.46(0.71) | 1.6(1.02) | -0.22 |
|  | Sotos | 2.91(2.11) | 3.01(1.7) | 2.91(2.21) | 2.77(1.57) | -0.05 |
| **d (loss-reward weight)** |  |  |  |  |  |  |
|  | FXS | 0.4(0.37) | 0.53(0.24) | 0.46(0.41) | 0.63(0.09) | -0.42 |
|  | CS | 0.61(0.36) | 0.67(0.31) | 0.71(0.43) | 0.65(0.43) | -0.18 |
|  | 22q11 | 0.68(0.26) | 0.63(0.18) | 0.67(0.28) | 0.59(0.15) | 0.21 |
|  | Sotos | 0.95(0.07) | 0.79(0.18) | 0.95(0.08) | 0.8(0.19) | 1.21 |

FXS = Fragile X syndrome; CS = Coffin Siris syndrome; Sotos = Sotos syndrome

**±** One participant with 22q11 deletion syndrome and two with Sotos syndrome lacked data from the social condition, resulting in a sample size of *n =* 5 and *n =* 2 respectively in these measures.

Long-term follow up

To validate the task, 10 participants (WS: n = 2, ID: n = 1, TD, n = 7) repeated the task in a research environment. The small sample size prevents meaningful statistical comparisons between time points, but visualizations of the data indicated highly similar values for choice behavior and computational modeling parameters between measurements (see *Figure S1*).

**
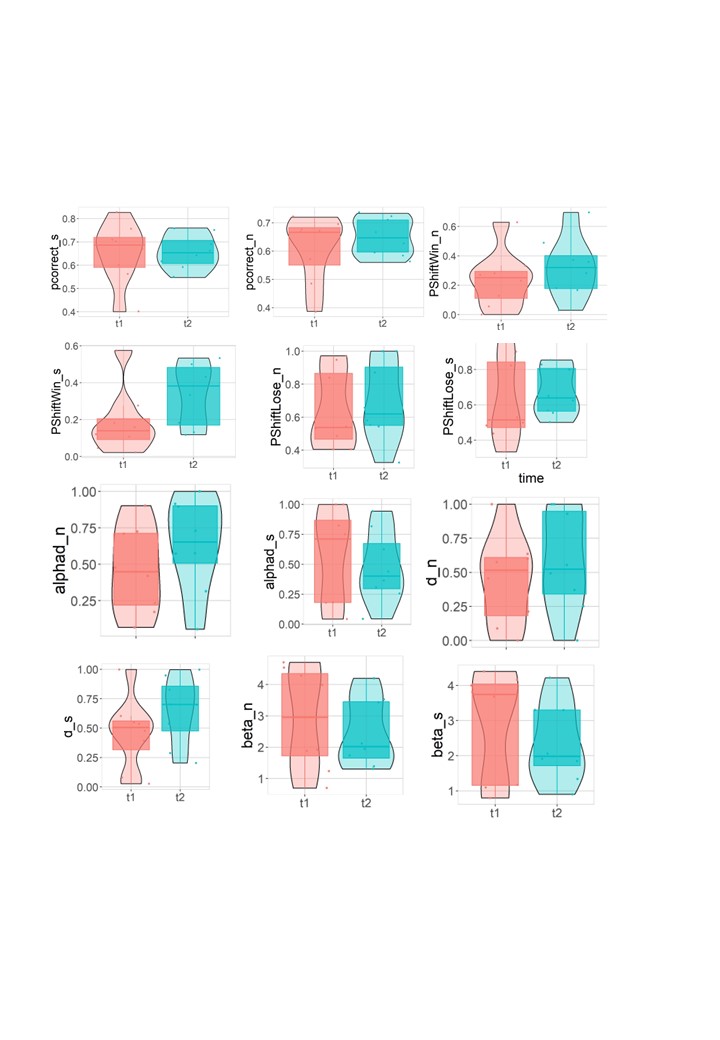
**

*Figure S1. Choice behavior results and computational modeling parameters per condition for participants who repeated the task at a later time point (n = 10, time to follow-up 2-7 months).* Boxplots cover medians and interquartile ranges. Violins show probability density functions. T1 = time-point 1. T2 = time-point 2 (repeated measurement).

**Development of correct choices over the course of the task**

**
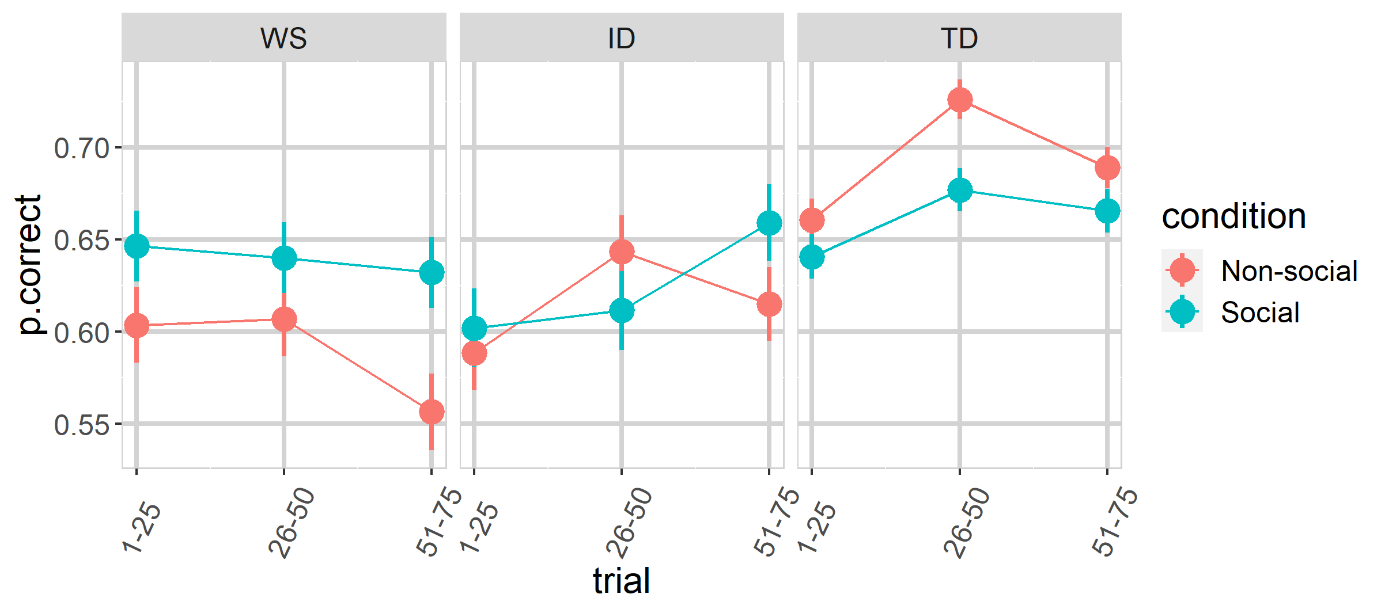
**

*Figure S2. Mean proportion of correct choices (p.correct) over blocks of 25 trials in the non-social and the social condition in the WS, ID, and TD groups. Error bars cover +/- 1 SE around the mean.*

Computational modeling analysis

We compared model fit to the data of several reinforcement learning and alternative models, introduced under *Overview of candidate models*. Models were further validated through data simulations.

Data were modeled at the level of the individual. Model comparison and parameter estimation were performed through maximum likelihood estimation using the *fminbnd* function in MATLAB. Following previous publications [1, 2], data were analyzed in two stages. First, for each model, the parameter values which maximized the log likelihood estimate (LLE) of the observed data were selected.

In stage two, this procedure was repeated with parameters restrained using Gaussian priors generated in stage one. Specifically, the log likelihood of the parameters under the prior distribution were added to the LLE of the observed parameters, a procedure which produces a priori more likely parameter values. This approach has been shown to increase model fit and reduce the risk of extreme parameter values [3].

Following previous publications[4], we computed individual Aikaike weights for the candidate models based on the Aikaike information criterion (AIC). The AIC is based on maximum likelihood estimation, but introduces a punishment for models with a higher number of free parameters[5]. The AIC for model *i* with *V* number of free parameters is calculated as:

-2* log(L_i_) + 2*V (*Eq. 1)*

Here, L_i_ is the maximum likelihood of the model. AIC values were transformed to Aikaike weights following [5]. These range from 0-1 and reflect the relative performance of each considered model relative to the others. To avoid local minima, maximum likelihood estimation was repeated 50 times for each individual and condition using randomly generated starting points and the values resulting in the highest likelihood were selected.

**Overview of candidate models**

***Model 1 (basic reinforcement learning model, RL***[6, 7]***)*** updates expected values of the choice made after each trial, based on the observed *prediction errors*, or the mismatch between expected and received rewards. The degree of updating is determined by the learning rate parameter α ranging between 0-1. A learning rate of 1 means that the expected value is always fully updated according to the most recent outcome, whereas a learning rate of 0 means that no learning takes place. Value updating is described by the equation:

$V{(c)}_{t+1}={V(c)}_{\begin{aligned} t \\ \end{aligned}}+ \alpha(r_{t}-{V\left( c \right)}_{t})$ (*Eq. 2)*

where *V(c)_t_* is the expected value of the chosen stimulus at trial *t*, *t* the trial number, *α* the learning rate, and r_t_ the reward received at trial *t*. Eq. 2 follows from the update rule for temporal difference learning applied to single-state Markov Decision Processes, also known as multi-armed bandit problems.

Expected values are transformed into choice probabilities with using a softmax equation:

$P(c_{t})=\frac{exp(\beta V(c_{t})}{exp(\beta V\left( c_{t} \right)+exp(\beta V\left( {nc}_{t} \right)}$ (*Eq. 3)*

where *P(c_t_)* is the probability of choosing stimulus *c at trial t*, V(c_t_) is the expected value of stimulus c, and *V(nc_t_)* the expected value of the other stimulus at trial t. The parameter $\beta$ ranging from 0 to infinity determines the degree of exploitation versus exploration. The probability of choosing the stimulus with a higher expected value increases with higher $\beta$. A $\beta$ of 0 means that choices are completely independent of expected values (*see Figure S1*). Model 1 therefore has two free parameters, α and β.


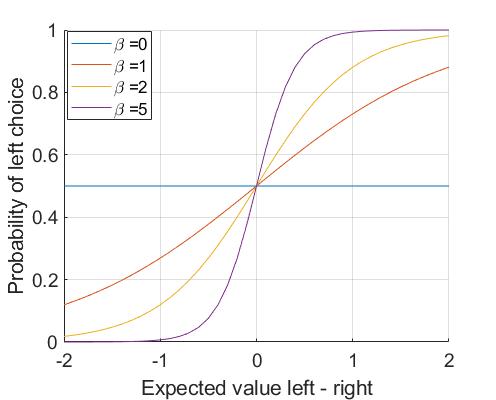


*Figure S3. Effects of different levels of* $\beta$ *on the probability of choosing the left over the right stimulus given differences in expected values plotted on the x-axis. Higher levels of* $\beta$ *lead to more deterministic choice probabilities (less exploration).*

***Model 2* (separate learning rate model, *RL2)*** has separate α for positive and negative outcomes, but is otherwise identical to the basic RL model (model 1).

***Model 3* (RL with reward/punishment sensitivity*)*** has a single α. Following previous studies [2, 8]the outcome value is determined by the free parameter *d* in the range [0,1]*,* so that

r = 1-d *(Eq.4)*

if the trial resulted in a win, and

r = -d *(Eq.5)*

if the trial resulted in a loss. The parameter *d* therefore indices the relative subjective value of rewards and losses, so that the relative weight given to punishments increases with higher values (henceforth referred to as loss/reward weight). Both outcomes are given equal weight if d = 0.5.

***Model 4* (*fictitious update reinforcement learning, fict.)*** is a modified version of the basic RL model (model 1) which updates the expected value of the chosen as well as the non-chosen action. Specifically, the value of the non-chosen action (nc) is updated according to:

$V{(nc)}_{t+1}={V(nc)}_{\begin{aligned} t \\ \end{aligned}}+ \alpha(1-r_{t}-{V\left( c \right)}_{t})$ *(Eq.6)*

Model 4 therefore has two free parameters ($\alpha$ and β).

***Model 5* (*fictitious update reinforcement learning + reward/punishment sensitivity balance)*** has a single learning rate $\alpha$, fictitious update of the non-chosen option, and the reward/punishment sensitivity parameter d described under model 3. As in models 1-4, Expected values are transformed into choice probabilities with using a softmax equation. Model 5 therefore has three free parameters ($\alpha$, β and d). Note that, when d = 0.5, models 5 and 4 make identical predictions.

***Model 6 (Choice kernel model, CK)*.** The choice kernel model [6]assumes that participants are not influenced by expected values or outcomes, but tend to repeat their previous choices. Therefore, instead of an expected value, the CK model updates a choice kernel which reflects how often a stimulus has been chosen previously. The choice kernel is updated according to the equation:

$CK{(c)}_{t+1}={CK(c)}_{\begin{aligned} t \\ \end{aligned}}+ \alpha(A_{t}-{CK\left( c \right)}_{t})$ (*Eq. 7)*

where CK(c) is the choice kernel value, *t* the trial, and $\alpha$ the learning rate. A is 1 if stimulus *c* was chosen, and otherwise 0. The learning rate $\alpha$ determines the degree to which the choice kernel is updated on each trial. As in model 1, choice probability is modeled with the softmax rule (*Eq. 2*). Model six therefore has two parameters, α and β.

***Model 7 (Alternating choices, alt)*.** Model 7 assumes that participants switch between the two choice alternatives at each trial with a probability of 1, regardless of observed outcomes. Model 7 does not have free parameters.

***Model 8 (Random responding, rand)*.** Model 8 assumes that participants choose at random, meaning that the probability of each choice is 0.5 throughout the experiment, regardless of observed outcomes. Model 8 does not have free parameters.

**Model selection**

Across groups, model 5 (fictitious update + reward/punishment sensitivity) had better fit to the data than the other models in both conditions (see *Figure S4*). The analysis was repeated in each group separately. As can be seen in *Figure S5*, model 5 had greater support in the WS and ID group. In the typically developed group, model 5 was slightly better in the social condition, whereas the model 1 had slightly better support in the non-social condition. For the sake of comparison, Model 5 was used in analyses in all groups.

**
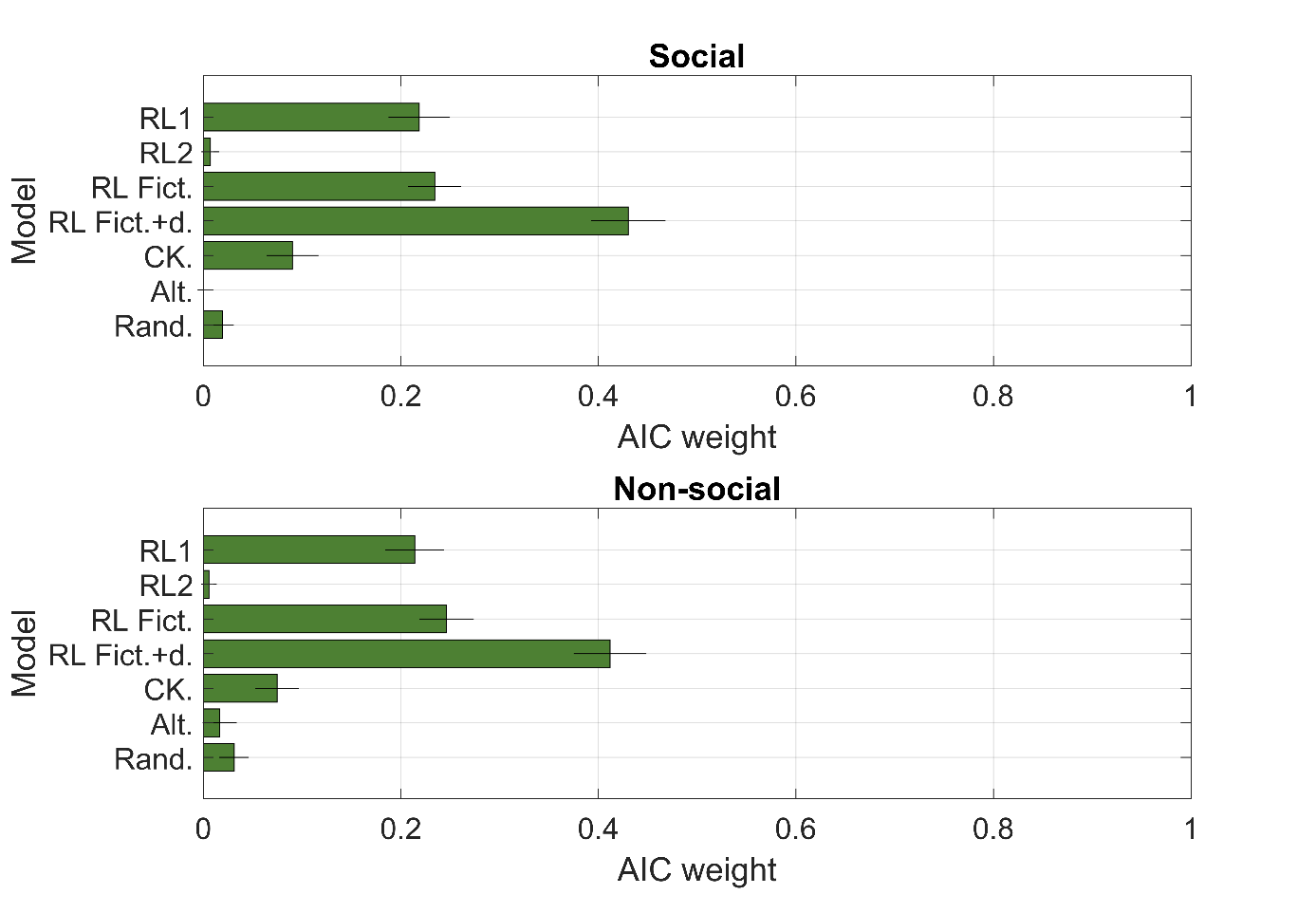
**

***Figure S4.*** AIC weights by condition for the candidate models.

**
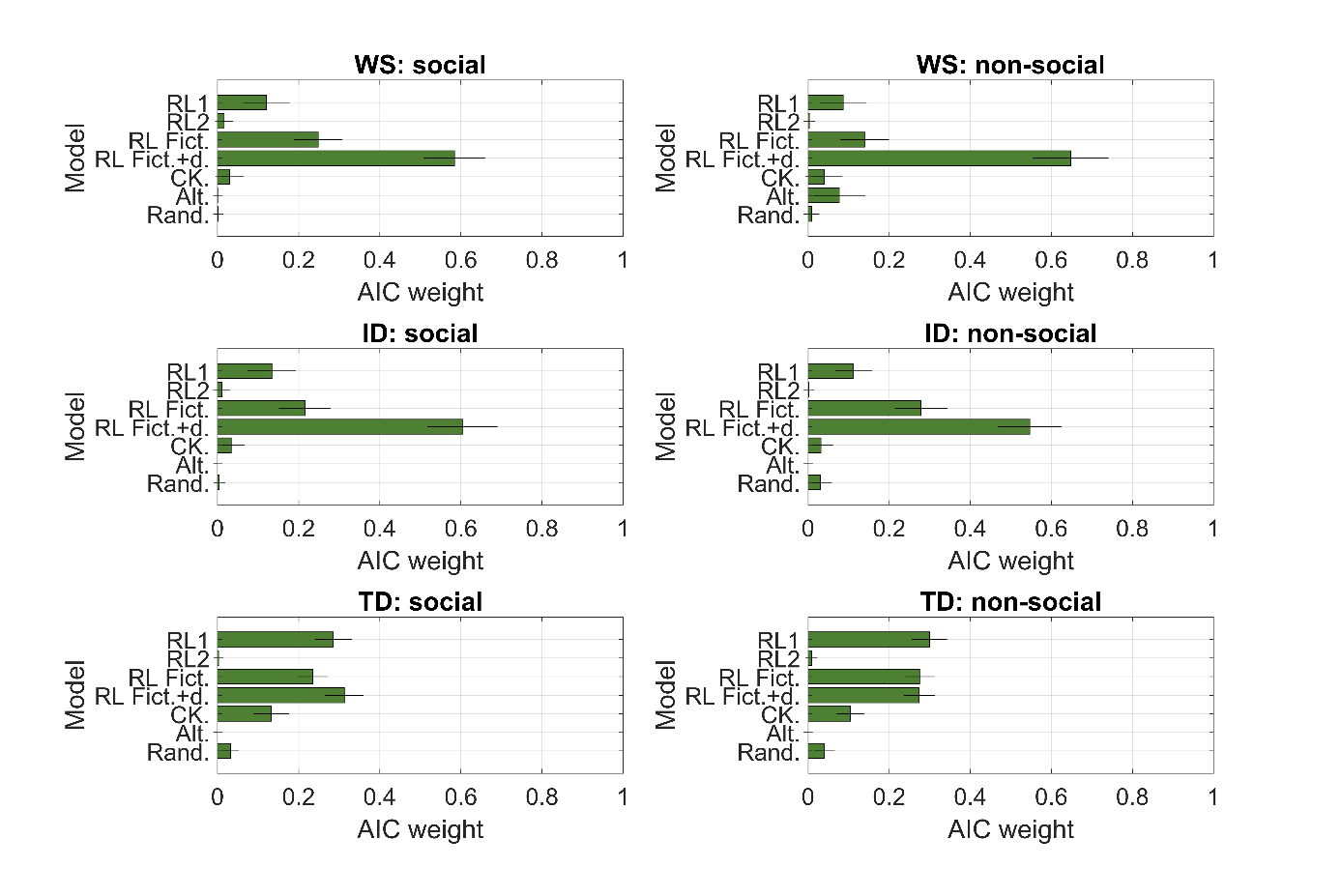
**

***Figure S5.*** AIC weights by condition for the candidate models in the WS, ID, and TD groups.

**Model validations**

For each participant and condition, we created 150 synthetic data sets where simulated agents completed the task using the winning models with parameter values of the actual participants. The choice behavior of these agents was then compared to the actual observed data. Results are shown in Figure S6A – B. As can be seen, the simulated data appears to capture the dynamics of observed choice behavior.

In a second step, we ran the main analyses (see *Results* in the manuscript), but for each participant replacing the observed data with the average of the 150 simulated data sets generated using their observed parameter values. All results were replicated in this synthetic data set, again suggesting that the selected model captured the relevant observed behavior.


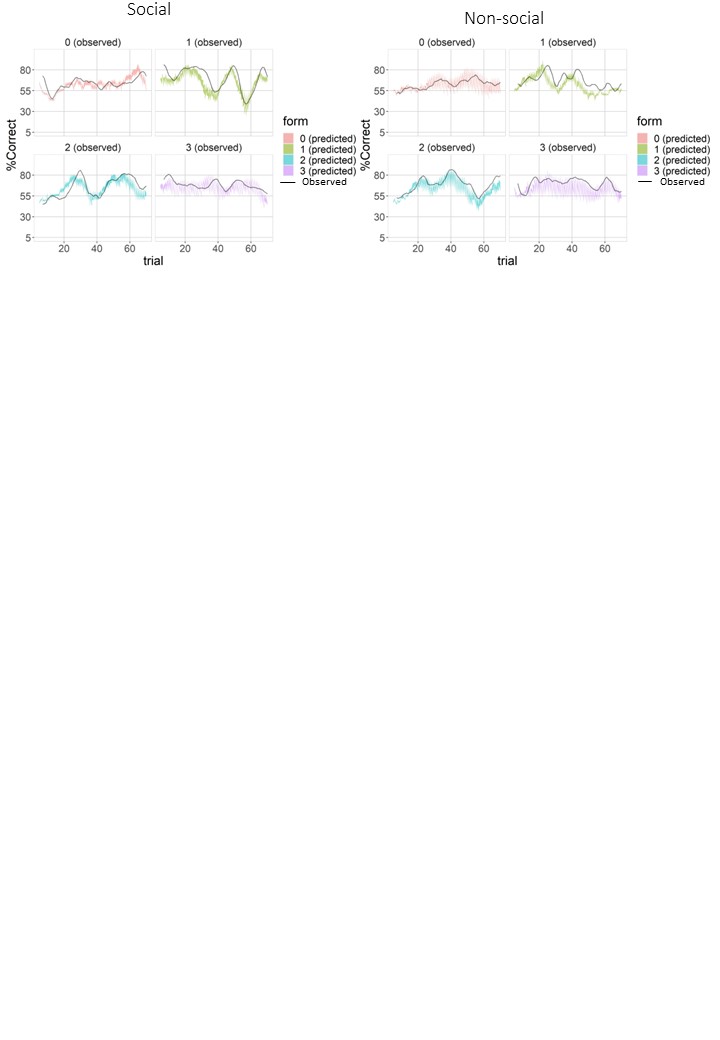


***Figure S6.*** *Observed choice behavior by group and in the social (left)* *and non-social (right) conditions compared to simulated data using the winning model with empirically derived parameter values (150 simulated data sets per participant). Forms 0 – 4 represent unique reinforcement schedules which were counterbalanced between groups and conditions. Shaded areas cover means and 95% confidence intervals of the simulated data.*

**Model recovery.** To examine whether candidate models could be distinguished in the observed data, we simulated a data set with 300 agents for each of the candidate models with parameter values in the 10-90^th^ percentile range of the observed data. Model fit of the candidate models to the simulated data was then compared using AIC weights. A confusion matrix showing the proportion of winning models in each of the simulated data sets is seen in *Figure S7*. As can be seen, the winning model could be distinguished from other models with high probability, with the exception of model 4 (RL Fict.). This is likely explained by the fact that the two models make identical predictions when *d* = 0.5, and are therefore a priori difficult to distinguish. The other candidate models were also adequately recovered, with the exception of models 2-3 which could not be distinguished from the RL1 and RL Fict. The non-learning models (Rand., Alt. and CK) were clearly distinguishable from the learning models.


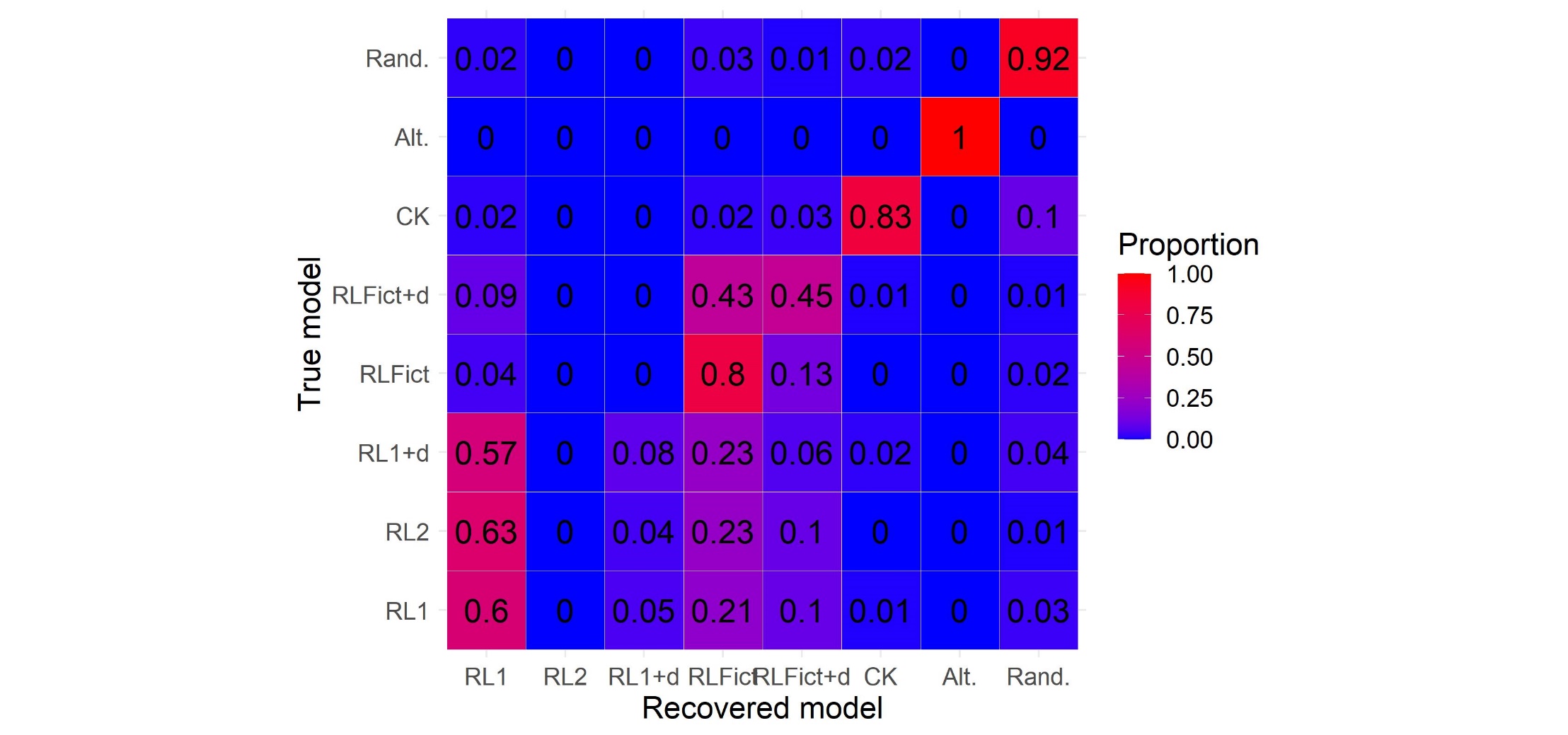


*Figure S7. Proportions of winning models in model comparisons of simulated data under each of the models.*

**Parameter recovery.** Correlations between simulated and recovered parameters were rs = .83 for α, rs =.76 for β and rs = .83 for *d* (see *Figur*e S8), indicating good parameter recovery.
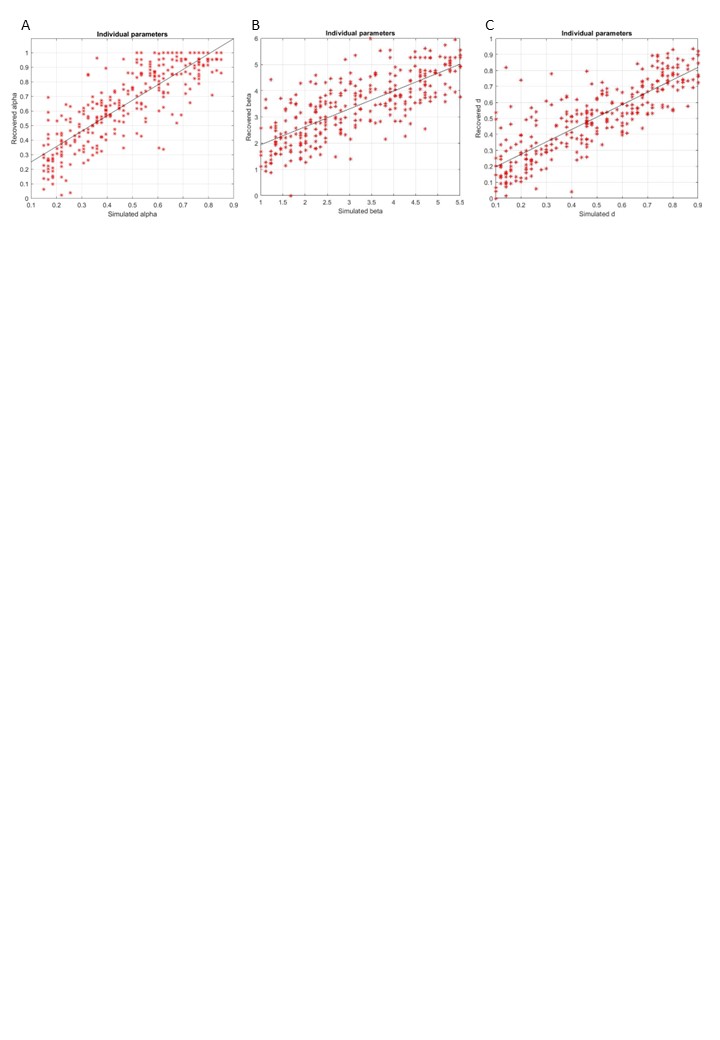


*Figure S8. Correlations between simulated and recovered parameter values for the winning model*

Reaction times

Descriptive statistics of reaction times by group and condition are shown in Table S1. Since data were positively skewed, a log-normal distribution was fitted on the reaction times for each participant and condition. The log-normal mu and sigma were used as measures of central tendency and variability. However, results remain unchanged when the median was analyzed instead.

*Log-normal mu.* A main effect of group was found (χ^2^(2) = 34.46, p <.001, f2 = 0.23), driven by quicker response times in TD than WS (χ^2^(1) = 37.88, p <.001, f2 = 0.32) and ID (χ^2^(1) = 29.77, p <.001, f2 = 0.24), whereas the WS and TD groups did not differ (χ^2^(1) = 0.02, p = .89, f2 < 0.01). The main effects of condition (χ^2^(2) = 0.36, p = .54, f2 < 0.01) and group x condition effects were non-significant (χ^2^(2) = 5.52, p = .063, f2 = 0.01).

*Reaction time variability (log-normal sigma).* Reaction times were also less variable in TD than WS (χ^2^(1) = 22.57 p <.001, f2 = 0.20) and ID (χ^2^(1) = 16.58, p <.001, f2 = 0.13), but the WS and ID groups did not differ (χ^2^(1) = 1.19, p = .275 f2 = 0.02). No main effect of condition χ^2^(2) = 0.59, p = .442, f2 < 0.01) or group x condition (χ^2^(2) = 0.39, p = .825, f2< 0.01) effects were found on reaction time variability.

*Table S2. Reaction times (lognormal mean, sigma, and median reaction time) by group and condition*

|  |  | Social condition | | Non-social condition | |
| --- | --- | --- | --- | --- | --- |
| Variable | Group | M (SD) | MD (MAD) | M (SD) | MD (MAD) |
| RT (lognormal mean) |  |  |  |  |  |
|  | WS | -0.08(0.63) | 0.06(0.73) | -0.09(0.66) | -0.24(0.75) |
|  | ID | -0.17(0.48) | -0.12(0.53) | 0.02(0.56) | -0.04(0.48) |
|  | TD | -0.57(0.48) | -0.71(0.34) | -0.7(0.39) | -0.75(0.37) |
| RT (lognormal sigma) |  |  |  |  |  |
|  | WS | 0.8(0.18) | 0.8(0.2) | 0.8(0.22) | 0.73(0.24) |
|  | ID | 0.77(0.13) | 0.75(0.14) | 0.74(0.18) | 0.72(0.2) |
|  | TD | 0.63(0.14) | 0.63(0.16) | 0.61(0.18) | 0.59(0.19) |
| RT (median) |  |  |  |  |  |
|  | WS | 1.1(0.88) | 1.01(0.76) | 1.1(0.83) | 0.71(0.45) |
|  | ID | 0.89(0.46) | 0.87(0.52) | 1.26(1.05) | 0.93(0.5) |
|  | TD | 0.63(0.48) | 0.45(0.16) | 0.49(0.24) | 0.42(0.14) |

WS = Williams syndrome; ID = Intellectual disability; TD = Typically developing; RT = reaction time

| **Measure** | **Group** | **Social (M, SD)** | **Non-social (M, SD)** |
| --- | --- | --- | --- |
| α |  |  |  |
|  | WS | 0.69(0.31) | 0.79(0.19) |
|  | ID | 0.69(0.3) | 0.71(0.23) |
|  | TD | 0.52(0.33) | 0.46(0.32) |
| β |  |  |  |
|  | WS | 2.88(1.2) | 2.76(0.88) |
|  | ID | 2.44(1.27) | 2.03(1.23) |
|  | TD | 2.26(1.07) | 2.5(1.15) |
| d |  |  |  |
|  | WS | 0.69(0.28) | 0.8(0.23) |
|  | ID | 0.63(0.33) | 0.67(0.26) |
|  | TD | 0.48(0.28) | 0.41(0.25) |

*Table S3. Descriptive statistics of computational modeling parameters by group and condition*

References

1. den Ouden HEM, Daw ND, Fernandez G, et al (2013) Dissociable Effects of Dopamine and Serotonin on Reversal Learning. Neuron 80:1090–1100. https://doi.org/10.1016/j.neuron.2013.08.030

2. Frey AL, Frank MJ, McCabe C (2019) Social reinforcement learning as a predictor of real-life experiences in individuals with high and low depressive symptomatology. Psychological Medicine. https://doi.org/10.1017/S0033291719003222

3. Daw ND (2011) Trial-by-trial data analysis using computational models. Decision making, affect, and learning: Attention and performance XXIII. In: Delgado MR, Phelps EA, Robbins TW (eds). Oxford University Press, Oxford

4. Stengard E, van den Berg R (2019) Imperfect Bayesian inference in visual perception. PLOS Computational Biology 15:e1006465. https://doi.org/10.1371/JOURNAL.PCBI.1006465

5. Wagenmakers E-J, Farrell S, Wagenmakers -J (2004) AIC model selection using Akaike weights. Psychonomic Bulletin & Review 11:192–196

6. Wilson RC, Collins AG (2019) Ten simple rules for the computational modeling of behavioral data

7. Sutton RS, Barto AG (2018) Reinforcement learning: An introduction. MIT Press

8. Gold JM, Waltz JA, Matveeva TM, et al (2012) Negative Symptoms and the Failure to Represent the Expected Reward Value of Actions Behavioral and Computational Modeling Evidence. Arch Gen Psychiatry 69:129–138
